# Oxyresveratrol and Gnetol Glucuronide Metabolites: Chemical Production, Structural Identification, Metabolism by Human and Rat Liver Fractions, and *In Vitro* Anti-inflammatory Properties

**DOI:** 10.1021/acs.jafc.1c07831

**Published:** 2022-02-23

**Authors:** Ruth Hornedo-Ortega, Michaël Jourdes, Gregory Da Costa, Arnaud Courtois, Julien Gabaston, Pierre-Louis Teissedre, Tristan Richard, Stéphanie Krisa

**Affiliations:** Unité de Recherche Œnologie, Institut des Sciences de la Vigne et du Vin, Université de Bordeaux, EA 4577, USC 1366 INRA, IPB, 210 Chemin de Leysotte, CS 50008, 33882 Villenave d’Ornon Cedex, France

**Keywords:** stilbenes, glucuronide, hemisynthesis, inflammation, metabolism

## Abstract

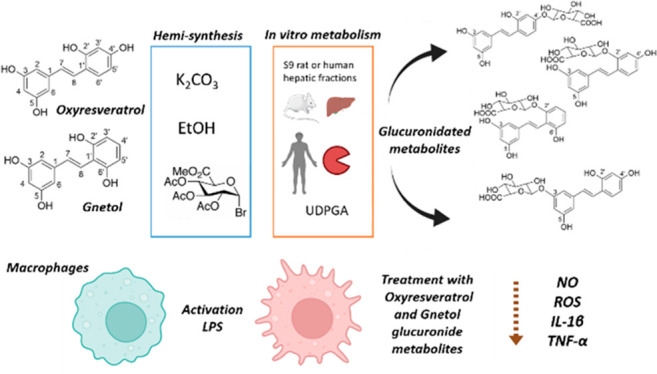

Stilbene metabolites are attracting great interest because many of them exhibit similar or even stronger biological effects than their parent compounds. Furthermore, the metabolized forms are predominant in biological fluids; therefore, their study is highly relevant. After hemisynthesis production, isolation, and structural elucidation, three glucuronide metabolites for oxyresveratrol (ORV) were formed: *trans*-ORV-4′-*O*-glucuronide, *trans*-ORV-3-*O*-glucuronide, and *trans*-ORV-2′-*O*-glucuronide. In addition, two glucuronide metabolites were obtained for gnetol (GN): *trans*-GN-2′-*O*-glucuronide and *trans*-GN-3-*O*-glucuronide. When the metabolism of ORV and GN is studied *in vitro* by human and rat hepatic enzymes, four of the five hemisynthesized compounds were identified and quantified. Human enzymes glucuronidated preferably at the C-2′ position, whereas rat enzymes do so at the C-3 position. In view of these kinetic findings, rat enzymes have a stronger metabolic capacity than human enzymes. Finally, ORV, GN, and their glucuronide metabolites (mainly at the C-3 position) decreased nitric oxide, reactive oxygen species, interleukin 1β, and tumor necrosis factor α production in lipopolysaccharide-stimulated macrophages.

## Introduction

**Figure 1:**
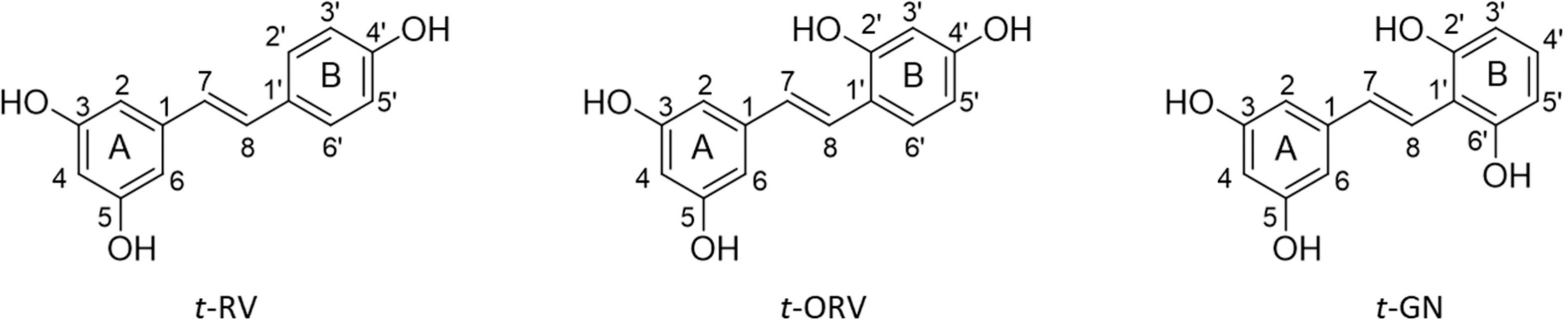
Structures of *trans*-resveratrol (*t*-RV), *trans*-oxyresveratrol (*t*-ORV), and *trans*-gnetol (*t*-GN).

Oxyresveratrol (ORV, *trans*-2′,3,4′,5-tetrahydroxystilbene) and gnetol (GN, *trans*-2′,3,5,6′-tetrahydroxystilbene) are stilbene monomers that are structurally very similar to resveratrol. The only difference between both compounds and resveratrol is the number and position of the two hydroxyl groups present on cycle B of the monomer (Figure 1). ORV is abundant in mulberry fruits (*Morus alba* L.) (21.7–106.7 mg/100 g) and in red, rosé, and white wines (1–5.4 μg/L).^1^ GN is abundant in plants of the genus *Gnetum* (0.11–3.76 μg/g), which is used in traditional medicine and in food products throughout Asia.^2^ However, whereas resveratrol is the most widely studied stilbene, a growing body of evidence is showing that GN and particularly ORV have potentially valuable bioactive properties.^3^ Both are attracting attention for human health, as confirmed by several *in vitro* and *in vivo* studies.^3^

ORV has been studied for its antioxidant, antimicrobial, antifungal, anti-inflammatory, and neuroprotective properties. It prevented amyloid β_25–35_-induced neuronal cell damage in rat cortical neurons by attenuating the increase in cytosolic Ca^2+^ levels and the release of glutamate and reducing reactive oxygen species (ROS).^4^ In addition, *in vivo* it reduced brain injury after cerebral stroke and attenuated neurological deficits by diminishing cytochrome *c* release and caspase 3 activation.^5^ Furthermore, using a macrophage cell line stimulated with lipopolysaccharide (LPS), ORV inhibited the production of nitric oxide (NO), nitric oxide synthase (iNOS), interleukin 6 (IL-6), and cyclooxygenase 2 (COX-2) mediated by the nuclear factor κB (NF-κB) signaling pathway.^6,7^ More recently, it proved active in preventing neuroinflammation in microglial cells.^8,9^*t*-ORV is able to suppress the release of NO, tumor necrosis factor α (TNF-α), inducible nitric oxide synthase (iNOS), interleukin 1β (IL-1β), monocyte chemoattractant protein 1 (MCP-1), C-X-C motif chemokine ligand 10 (CXCL10), and IL-6 via NF-κB but also via the mitogen-activated protein kinase (MAPK) and PI3K/AKT/p70S6K signaling pathways.^8^ With regard to GN, it has been found to be a potent tyrosinase and acetylcholinesterase inhibitor.^10,11^ Moreover, it significantly reduced cell viability in cancer cell lines (mainly in colorectal cancer cells) and inhibited platelet aggregation or platelet–collagen adhesion and the enzymatic activity of cyclooxygenase 1 (COX-1).^2,12^

Even though several biological activities have been attributed to stilbenes, they are poorly absorbed by the intestines and are highly metabolized. After absorption, they are conjugated to glucuronide, sulfate, and methyl groups in the gut mucosa and inner tissues. Uridine 5′-diphosphate glucuronide transferase, sulfotransferase, and catechol *O*-methyltransferase are phase II metabolism enzymes that are responsible for these reactions. For this reason, non-conjugated stilbenes are practically absent in plasma. Moreover, when stilbenes reach the colon, they are broadly metabolized by microflora, resulting in other transformed compounds.^13^ Finally, microbial metabolites may be absorbed and reach the liver as well as other tissues for further metabolism or excretion.^14^

The bioavailability of stilbenes is extremely different from one molecule to another. Some monomers are bioavailable, while most oligomers are only weakly available.^15^ In general, when the degree of oligomerization increases, bioavailability decreases.^15^ The total bioavailability of ORV has been reported to be between 9.1 and 15.2% depending upon the dose (100, 200, or 400 μmol/kg).^16^ Glucuronide, methylate, and sulfate metabolites have been detected in bile, urine, and plasma.^17−19^ Around a 10 μM concentration of ORV has been quantified in plasma after the oral administration of ORV in rats (100 mg/kg).^19^ Although the micromolar concentration of ORV glucuronides was not described in this paper, the AUC_0–*t*_ values for ORV and its glucuronides (5133 versus 10518 μg h L^–1^) suggest that the conjugates are more present than the aglycone.^19^ Concerning GN, its calculated bioavailability after intravenous and oral administration in rats is 6.6%.^2^ In two published studies on the pharmacokinetics of GN, these same authors presented only the AUC values of GN. However, their results showed that glucuronides were predominantly present.^2,20^ Hence, data about physiological plasma concentrations of ORV and GN glucuronide metabolites are scarce. Nevertheless, it is well-established that plasma concentrations of glucuronide metabolites are higher (2–100 more times) than their parent compounds, as stated for other stilbenes.^21,22^

While the biological activities of stilbenes have been investigated for several decades, their metabolites are attracting much attention because many of them exhibit very similar or even higher biological effects than their parent compounds. Furthermore, the metabolized forms are predominant in biological fluids and various tissues; therefore, it is relevant to investigate their effects. While the glucuronide and sulfate metabolites of resveratrol have been well-characterized and are commercially available,^23^ metabolites of other stilbenes are lacking or are costly; therefore, chemical synthesis in the laboratory is the only way to produce their metabolites.

We therefore sought to achieve the chemical hemisynthesis of ORV and GN glucuronides and their structural elucidation for the first time using nuclear magnetic resonance (NMR) techniques. After pure glucuronide metabolites of ORV and GN were obtained, the metabolism of ORV and GN by human and rat liver microsomes was assessed *in vitro*. Finally, the potential anti-inflammatory and antioxidant [NO, IL-1β, TNF-α, and intracellular ROS production] properties of ORV and GN and their synthesized glucuronide metabolites were assessed in LPS-stimulated macrophages (RAW 264.7 cell line).

## Materials and Methods

### Standards and Reagents

ORV and GN were purchased from TCI Chemicals (Zwijndrecht, Belgium). Acetobromo-α-d-glucuronic acid methyl ester, uridine 5′-diphosphoglucuronic acid trisodium salt (UDPGA), MgCl_2_, Tris–HCl, LPS, Roswell Park Memorial Institute (RPMI) medium, Dulbecco’s modified Eagle’s medium (DMEM), fetal bovine serum (FBS), Griess reagent, 2′,7′-dichlorodihydrofluorescein diacetate acetyl (DCFH_2_-DA), 3-(4,5-dimethyl-2-thiazolyl)-2,5-diphenyl-2*H*-tetrazolium bromide (MTT), trifluoroacetic acid (TFA), dimethyl sulfoxide (DMSO), and glutamine were obtained by Sigma-Aldrich (Steinheim, Germany). Alamethicin was purchased from Santa Cruz Biotechnology (Heidelberg, Germany). Male rat and human hepatic S9 fractions were obtained by Biopredic (Saint Grégoire, France). RAW 264.7 cells were provided by American Type Culture Collection (ATCC, Manassas, VA, U.S.A.). Mouse TNF-α enzyme-linked immunosorbent assay (ELISA) was purchased by InmunoTools (Friesoythe, Germany), and mouse IL-1β ELISA was provided by BioLegend (San Diego, CA, U.S.A.).

### Production of GN and ORV Glucuronide Metabolites by Hemisynthesis

Monoglucuronides of GN and ORV were obtained by chemical *O*-glucuronidation using acetobromo-α-d-glucuronic acid methyl ester in alkaline conditions.^24^ To optimize the method, different bases (KOH, NaOH, and K_2_CO_3_), incubation times (1, 2, 4, 8, 24, and 48 h), and equivalences of ORV, GN, acetobromo-α-d-glucuronic acid methyl ester, and bases were tested.

### Ultra Performance Liquid Chromatography–Diode Array Detection/Electrospray Ionization Quadrupole Time-of-Flight (UPLC–DAD/ESI-Q-TOF) Analysis

Prior to purification, 5 μL of samples from GN and ORV hemisynthesis reactions (50 μL in 500 μL of H_2_O/formic acid) was injected in an UPLC–DAD/ESI-Q-TOF system (Agilent 1290 Infinity Agilent Technologies, Santa Clara, CA, U.S.A.) equipped with ultraviolet–visible (UV–vis) DAD and an ESI-Q-TOF mass spectrometer (Agilent 6530 Accurate Mass) to identify and obtain the exact mass of produced glucuronide metabolites. Analysis was carried out on an Agilent Zorbax SB-C18 (100 mm × 2.1 mm × 1.8 μm) column. Separation was performed with a solvent system consisting of solvent A (water with 0.1% formic acid) and solvent B (methanol with 0.1% formic acid). Separation was performed using a flow rate of 0.3 mL/min. The run was as follows: 6–50% B (from 0 to 20 min), 50–100% (from 20 to 25 min), and 100% (from 25 to 30 min). Mass spectrometry analyses were performed in negative mode. The drying gas used was nitrogen at 9 L/min and 300 °C with nebulizer pressure at 25 psi. The sheath gas flow and temperature were set at 11 L/min and 350 °C. Capillary voltage was 4000 V. The data were processed by Mass Hunter Qualitative Analysis software (version B0800).

### Preparative High-Performance Liquid Chromatography (HPLC)

The obtained solution after hemisynthesis was evaporated and resuspended in 2–3 mL of Milli-Q water prior to purification using a preparative HPLC system (PLC 2050, Gilson). Separation was carried out using a Phenomenex Kinetex 100-5 XB-C18 (5 μm, 150 × 21.2 mm) column. Water with 0.005% TFA (solvent A) and acetonitrile with 0.005% TFA (solvent B) were used as elution solvents. The flow rate was set at 20 mL/min, and the UV detector was set at wavelengths of 280 and 320 nm. The gradient was as described: 10% B (from 0 to 10 min), 10–30% B (from 10 to 25 min), 30–100% B (from 25 to 30 min), 100% B (from 30 to 35 min) 100–10% B (from 35 to 36 min), and 10% B (from 36 to 41 min). Each compound was then evaporated and injected in the UPLC–DAD/ESI-Q-TOF system, as explained above, to control the purity.

### Structural Identification of GN and ORV Metabolites by NMR

All one-dimensional (1D) and two-dimensional (2D) NMR experiments were performed on a Bruker Avance 600 MHz NMR spectrometer (Bruker, Wissembourg, France) operating at 600.3 MHz and equipped with a 5 mm TXI probe. Data were processed using TopSpin software, version 3.2 (Bruker BioSpin, Germany). 1D and 2D NMR spectra were acquired in methanol-*d*_4_ at 293 and 303 K, with 1.5 s relaxation time. All 2D experiments were carried out with 2048 data points × 400 increments, using spectral widths of 8417 and 33 209 Hz in proton and carbon dimensions, respectively. Mixing time was 300 ms and the spinlock time was 100 ms for rotating-frame nuclear Overhauser effect spectroscopy (ROESY) and total correlation spectroscopy (TOCSY) experiments, respectively.

### *In Vitro* Metabolism of ORV and GN by Human and Rat Hepatic S9 Fractions

To study the production of glucuronide metabolites *in vitro*, human and rat S9 hepatic fractions were used. To ensure that the formation rates of metabolites were linear over the incubation time and at the concentration of protein, a series of preliminary experiments was performed to optimize the reactions.

Glucuronidation was studied by incubation of human or rat liver S9 fractions (0.5 mg/mL) with ORV or GN at different concentrations (0–3000 μM) in Tris–HCl buffer (50 mM, pH 7.4) in the presence of UDPGA (1 mM), alamethicin (25 μg/mL), and MgCl_2_ (5 mM). All samples were prepared in Eppendorf tubes in a final volume of 100 μL. After 15 min at 37 °C, 100 μL of methanol (100%) was incorporated to stop the reaction and precipitate the proteins. Finally, samples were centrifuged for 30 min at 14000*g* and 4 °C, and the supernatants were analyzed by UPLC–DAD–MS. Analysis were carried out using 1290 Infinity UPLC (Agilent Technologies, Courtaboeuf, France). The UPLC system was coupled to an Esquire 3000 Plus ion trap mass spectrometer using an ESI source (Bruker-Daltonics, Billerica, MA, U.S.A.). A total of 2 μL was injected into an Agilent SB-C18 column (1.8 μm, 2.1 × 100 mm). Samples were eluted with solvent A (H_2_O/0.1% formic acid) and solvent B (acetonitrile/0.1% formic acid) with the following gradient program: 10% B (from 0 to 1.7 min), 10–20% B (from 1.7 to 3.4 min), 20–30% B (from 3.4 to 5.1 min), 30% B (from 5.1 to 7.8 min), 30–35% B (from 7.8 to 9.5 min), 35–60% B (from 9.5 to 11.9 min), 60–100% B (from 11.9 to 15.3 min), 100% B (from 15.3 to 17 min), and 100–10% B (from 17 to 17.3 min). The flow rate was set at 0.4 mL/min, and the UV detector was set at wavelengths of 280 and 320 nm. Total ion chromatograms were obtained using negative mode with a range of *m*/*z* 130–1400. The parameters were as follows: capillary voltage, +4 kV; nebulizer pressure, 40 psi; dry gas, 10 L/min; and dry temperature, 365 °C. Data analysis was performed with Bruker Data Analysis 3.2 (Bruker-Daltonics, Billerica, MA, U.S.A.). Metabolite concentrations were quantified with the corresponding standard curve of ORV and GN glucuronides previously obtained by hemisynthesis.

### Cell Culture of RAW 264.7 and Treatment with GN, ORV, and Their Metabolites

RAW 264.7 cells were cultured in DMEM containing 10% FBS and maintained in a humidified incubator set at 37 °C with 5% CO_2_. For all following assays, cells were subcultured at a density of 40 000 cells per well in 96 well culture plates with 200 μL of the above-described culture medium. After 24 h, cells were incubated with ORV, GN, and their glucuronide metabolites (5–200 μM) in RPMI medium supplemented with glutamine (4 mM) in the presence or absence of LPS (0.1 μg/mL) (200 μL final volume per well). For MTT, NO, ROS, IL-1β, and TNF-α (*n* = 4), each experiment was performed in triplicate separately.

### MTT Cell Viability

The MTT colorimetric assay was performed to assess the cell viability of cells after being treated with ORV, GN, and their glucuronide metabolites. After 24 h of treatment, RAW 264.7 cells were incubated with 0.5 mg/mL MTT during 3 h at 37 °C. The formazan crystals formed were dissolved with 100 μL of dimethyl sulfoxide (DMSO), and the plate was then incubated in the darkness for 30 min. Finally, the absorbance was measured at 595 nm using a microplate reader (FLUOstar Optima, BMG Labtech).

### Intracellular NO Measurement

After 24 h of treatment of RAW 264.7 cells with ORV, GN, and their glucuronide metabolites, 70 μL of supernatant was mixed with 70 μL of Griess solution. After 15 min in darkness, the absorbance was measured at 550 nm using a microplate reader (FLUOstar Optima, BMG Labtech). A calibration curve of NO_2_ (0–100 μM) was used for quantification. Data were expressed as NO production (μM) compared to cells treated only with LPS (positive control).

### Intracellular ROS Measurement

Generation of intracellular ROS in cells was analyzed using the fluorometric probe DCFH_2_-DA. After 24 h of treatment with GN, ORV, and their glucuronide metabolites, cells were washed with phosphate-buffered saline (PBS) and then 150 μL of DCFH_2_-DA (10 μM) was added. After 30 min at 37 °C, the fluorescence intensity was quantified using a microplate reader (FLUOstar Optima, BMG Labtech) with a wavelength of excitation and emission of 485 and 520 nm, respectively. All experiments were performed in darkness. Results were expressed as ROS production (fluorescence intensity expressed as arbitrary units) compared to cells treated only with LPS (positive control).

### Measurement of TNF-α and IL-1β Production (ELISA Assay)

At 24 h after seeding, RAW 264.7 cells were exposed to LPS (0.1 μg/mL) in the absence or presence of ORV, GN (10 and 50 μM), and their metabolites (50 and 200 μM). After 24 h of exposure, IL-1β and TNF-α concentrations were measured in culture media supernatants by ELISA sandwich assay following the instructions of the manufacturer.

### Data Analysis

The kinetic parameters, *K*_m_, *V*_max_, and *K*_i_, were calculated using the GraphPad Prism software (version 6.01) (substrate inhibition as the kinetic model). The data obtained from MTT, NO, ROS, IL-1β, and TNF-α production were subjected to one-way analysis of variance (ANOVA). A comparison between LPS and the different tested concentrations of ORV, GN, and their glucuronide metabolites was performed using Dunnet’s test, and *p* < 0.05 was considered significant (GraphPad Prism software, version 6.01).

## Results and Discussion

### Hemisynthesis and Structural Elucidation of ORV and GN Glucuronide Metabolites by NMR and UPLC–DAD/ESI-Q-TOF

**Figure 2:**
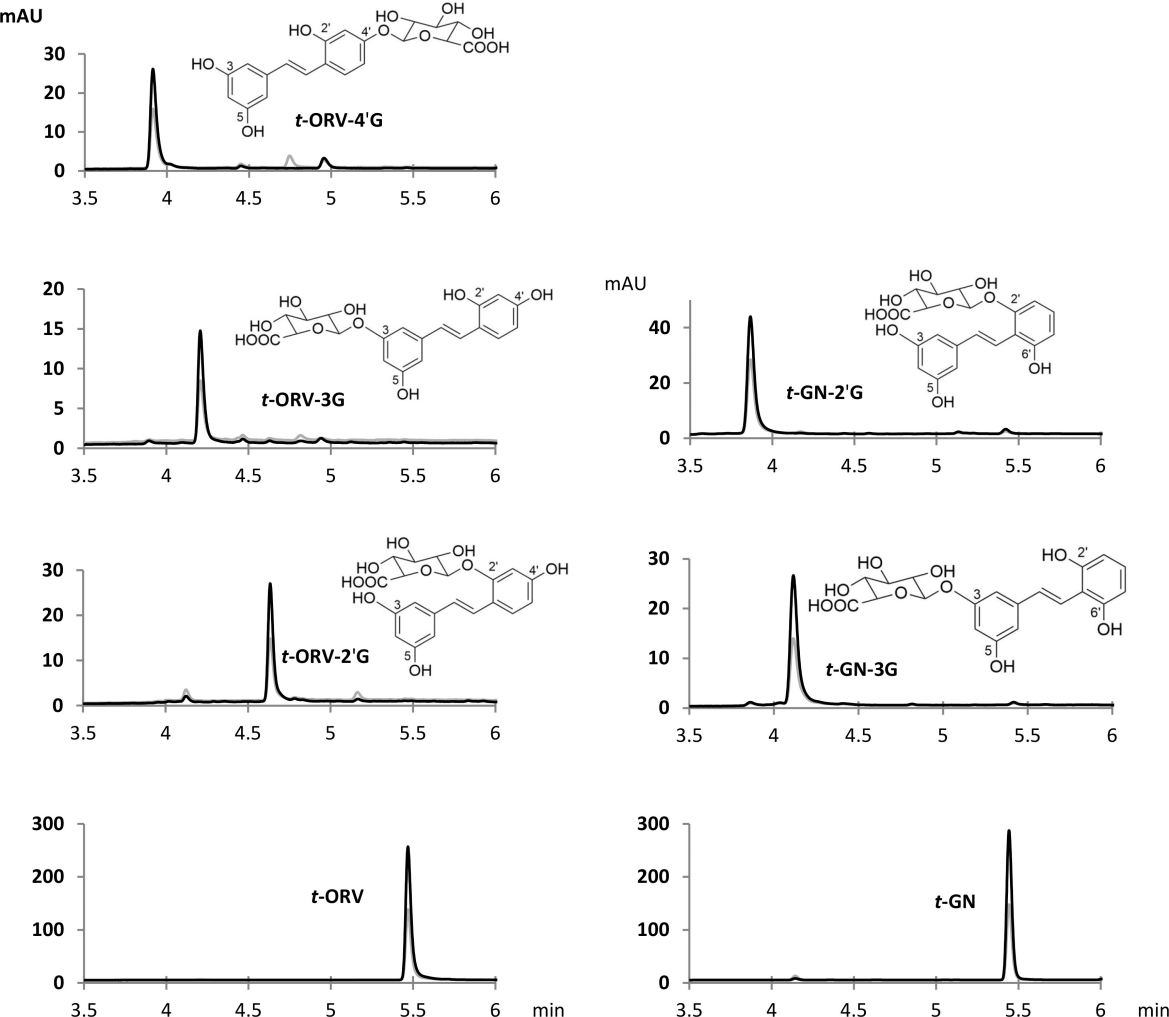
Structure and UHPLC–DAD chromatograms at 280 nm (gray line) and 320 nm (black line) of ORV and GN glucuronide metabolites obtained by hemisynthesis. *t*-ORV-4′G, *trans*-oxyresveratrol-*O*-4′-glucuronide; *t*-ORV-3G, *trans*-oxyresveratrol-*O*-3-glucuronide; *t*-ORV-2′G, *trans*-oxyresveratrol-*O*-2′-glucuronide; *t*-ORV, *trans*-oxyresveratrol; *t*-GN-2′G, *trans*-gnetol-*O*-2′-glucuronide; *t*-GN-3G, *trans*-gnetol-*O*-3-glucuronide; and *t*-GN, *trans*-gnetol.

The absence of commercial standards and the need to obtain sufficient quantities for characterization purposes as well as to study their anti-inflammatory properties make hemisynthesis from the parental monomer the best option. The optimized hemisynthesis reaction to obtain glucuronide conjugate metabolites was carried out as follows: ORV or GN (50 mg, 1 equiv) was dissolved in ethanol (10 mL), and K_2_CO_3_ (30 mg/mL, 5 equiv) was added. Then, acetobromo-α-d-glucuronic acid methyl ester (2 equiv) dissolved in 10 mL of ethanol was incorporated in the reaction vial. This was placed in a stove set at 50 °C for 48 h. Thereafter, the solution was neutralized with acidified water to reach a pH of 5.5. Various hemisynthesis reactions followed by HPLC preparative purifications were carried out to obtain each glucuronide metabolite (10% yield). Structural determinations were established by combining NMR and UPLC–DAD/ESI-Q-TOF analyses. The hemisynthesis of ORV led to the formation of three main derivatives. UV chromatograms and structures are displayed in Figure 2. The mass spectra of these compounds exhibited a molecular ion at *m*/*z* 419.0807 and a specific fragment ion at *m*/*z* 243.0578 corresponding to the loss of the glucuronic acid moiety (*m*/*z* 176.0229). Structural determination was achieved by 1D and 2D NMR spectroscopy. ^1^H NMR data are shown in Table 1.

**Table 1:** ^1^H NMR Data for ORV and GN Glucuronides

number	*t*-ORV	*t*-ORV-4′G	*t*-ORV-3G	*t*-ORV-2′G	*t*-GN	*t*-GN-2′G	*t*-GN-3G
2	6.45 d (2)	6.47 d (2)	6.73 brs	6.51 d (2)	6.48 d (2)	6.52 d (2)	6.75 brs
3							
4	6.14 t (2)	6.16 t (2)	6.43 brs	6.16 t (2)	6.15 t (2)	6.16 t (2)	6.45 brs
6	6.45 d (2)	6.47 d (2)	6.65 brs	6.67 d (2)	6.48 d (2)	6.52 d (2)	6.67 brs
7	6.83 d (16)	6.91 d (16)	6.88 d (16)	6.81 d (16)	7.4 d (16)	7.52 d (16)	7.44 d (16)
8	7.28 d (16)	7.30 d (16)	7.31 d (16)	7.53 d (16)	7.48 d (16)	7.55 d (16)	7.52 d (16)
3′	6.31 brs	6.59 brs	6.32 brs	6.51 d (2)	6.36 d (8)	6.71 d (8)	6.35 d (8)
4′					6.85 t (8)	7.00 t (8)	6.86 t (8)
5′	6.32 dd (2, 9)	6.60 dd (2, 9)	6.32 dd (2, 9)	6.54 dd (2, 9)	6.36 d (8)	6.58 d (8)	6.35 d (8)
6′	7.34 d (9)	7.45 d (9)	7.35 d (9)	7.49 d (9)			
Glucuronide Moiety							
G1		4.95 d (7)	4.97 d (7)	4.93 d (7)		5.01 d (8)	4.98 d (7)
G2		3.50–3.70	3.50–3.70	3.50–3.70		3.50–3.70	3.50–3.70
G3		3.50–3.70	3.50–3.70	3.50–3.70		3.50–3.70	3.50–3.70
G4		3.50–3.70	3.50–3.70	3.50–3.70		3.50–3.70	3.50–3.70
G5		3.99 d (10)	3.99 d (10)	3.93 d (10)		3.96 d (10)	4.00 brs

ORV glucuronides were identified on the basis of nuclear Overhauser effect spectroscopy (NOESY) correlations with chemical shift variation and were confirmed by heteronuclear multiple-bond correlation (HMBC) experiments. The first compound with a retention time at 3.9 min showed nuclear Overhauser effect (NOE) correlations between the anomeric proton H-G1 and protons H-3′ and H-5′, indicating that glucuronic acid is attached to the C-4′ carbon. Thus, this compound was identified as *trans*-ORV-4′-*O*-glucuronide (*t*-ORV-4′G). In addition, the chemical shifts of protons H-3′ and H-5′ were shifted upfield compared to those of *trans*-ORV. These data were confirmed by HMBC spectra. The second ORV metabolite with a retention time of 4.2 min corresponding to *trans*-ORV-3-*O*-glucuronide (*t*-ORV-3G) exhibited NOE correlations between the anomeric proton H-G1 and the protons H-2 and H-4, indicating that glucuronic acid is attached to the C-3 carbon. This hypothesis was confirmed by the chemical shift variations of H-2 and H-4 protons in comparison to *trans*-ORV. Finally, the third compound with a retention time of 4.6 min was identified as *trans*-ORV-2′-*O*-glucuronide (*t*-ORV-2′G) based on NOE correlation between anomeric proton H-G1 and the aglycone signal corresponding to proton H-3′. In addition, the H-3′ proton was shifted upfield compared to that of *t*-ORV. These data indicate that glucuronic acid is attached to the C-2′ carbon.

With regard to GN derivatives, two compounds were formed and identified (Figure 2). The mass spectra exhibited a molecular ion at *m*/*z* 419.0809 and a specific fragment ion at *m*/*z* 243.0576, corresponding to the loss of the glucuronic acid moiety. ^1^H NMR data are shown in Table 1. On the basis of NOESY spectra and chemical shift variations, the first compound with a retention time of 3.8 min was identified as *trans*-GN-2′-*O*-glucuronide (*t*-GN-2′G) and the second compound with a retention time of 4.1 min was identified as *trans*-GN-3-*O*-glucuronide (GN-3G). The first compound exhibited a NOE correlation between the anomeric proton (H-G1) and the H-3′ proton, indicating that glucuronic acid is attached to the C-2′ carbon. The second compound presented NOE correlations between the anomeric proton (H-G1) and protons H-2 and H-4, indicating that glucuronic acid is attached to the C-3 carbon. These hypotheses were confirmed by chemical shift variations and HMBC spectra. Thanks to hemisynthesis, we therefore report for the first time the unambiguous structural identification of three ORV and two GN glucuronide metabolites.

### Profile and Kinetic Parameters of Human and Rat Glucuronide Metabolites of ORV and GN

An important objective of this work was to study the *in vitro* metabolization of *t*-ORV and *t*-GN in rat and human species using S9 hepatic fractions. To this end, both stilbene monomers were incubated (37 °C, 15 min) at different concentrations (0–3000 μM) with human and rat S9 fractions (0.5 mg/mL) in the presence of UDPGA as a co-substrate of phase II enzymes. Then, samples were injected into a HPLC–DAD–MS system to identify and quantify the *in vitro* generated metabolites.

**Figure 3:**
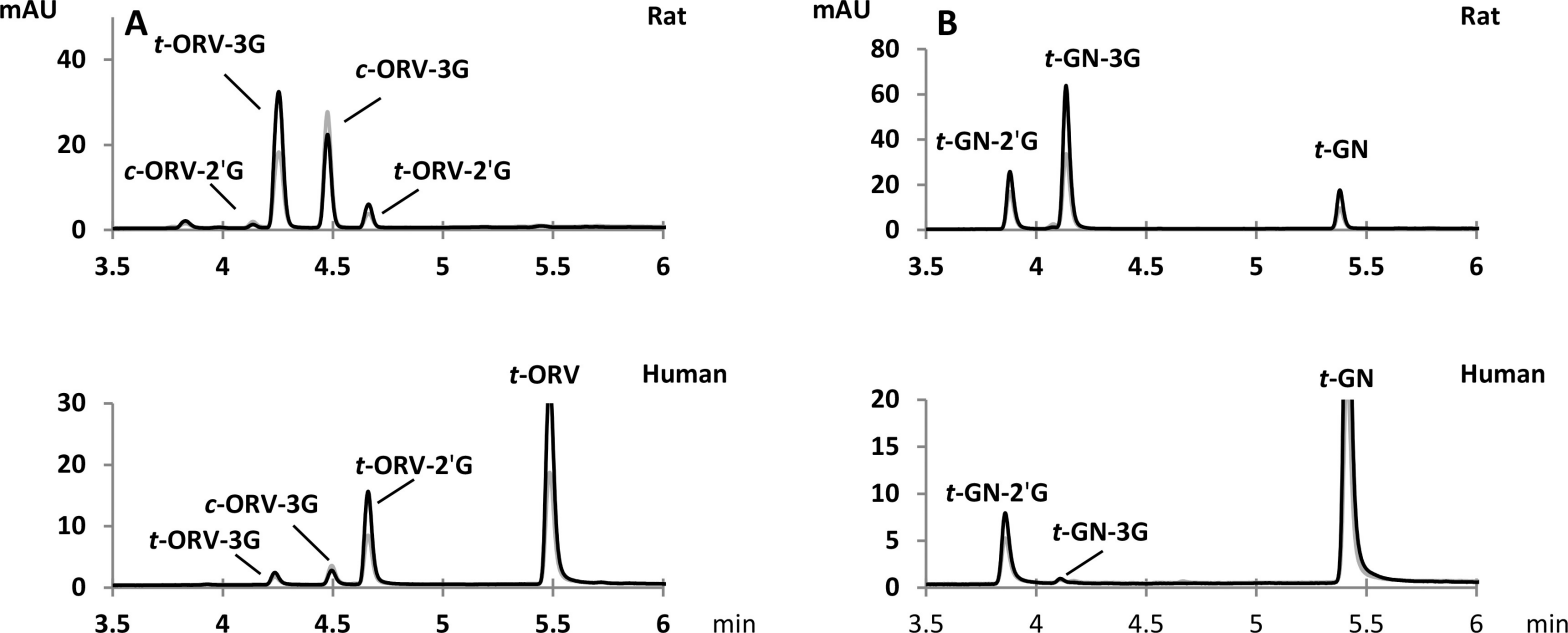
UHPLC–DAD chromatograms at 280 nm (gray line) and 320 nm (black line) of (A) ORV and its metabolites and (B) GN and its metabolites obtained by metabolization (at 50 μM ORV and GN). The upper part corresponds to rat protein, and the lower part corresponds to human protein. *t*-ORV-3G, *trans*-oxyresveratrol-*O*-3-glucuronide; *c*-ORV-3G, *cis*-oxyresveratrol-*O*-3-glucuronide; *t*-ORV-2′G, *trans*-oxyresveratrol-*O*-2′-glucuronide; *c*-ORV-2′G, *cis*-oxyresveratrol-*O*-2′-glucuronide; *t*-ORV, *trans*-oxyresveratrol; *t*-GN-2′G, *trans*-gnetol-*O*-2′-glucuronide; *t*-GN-3G, *trans*-gnetol-*O*-3-glucuronide; and *t*-GN, *trans*-gnetol.

Two of the three monoglucuronide ORV metabolites obtained by hemisynthesis were identified in both human and rat samples: *trans*-ORV-3-*O*-glucuronide (*t*-ORV-3G) and *trans*-ORV-2′-*O*-glucuronide (*t*-ORV-2′G) (Figure 3). To quantify these compounds, the UV spectra at 320 nm and the calibration curves of purified compounds were used. After a detailed analysis of UV data, we noted two more peaks that absorbed more at 280 nm than at 320 nm. *cis*-Stilbenes are known to possess a λ_max_ close to 280 nm, while the *trans* form exhibits a λ_max_ between 306 and 325 nm.^25^ Therefore, we considered the possible isomerization of the *trans* to *cis* form. To settle any doubts, we exposed the three chemically hemisynthesized metabolites to an UV lamp (set at 365 nm) for 1 h to induce isomerization.^26^ The compounds present in the samples were then analyzed by UPLC–DAD–MS. We thus confirmed the presence of two *cis* metabolites: *cis*-ORV-3-*O*-glucuronide (*c*-ORV-3G) and *cis*-ORV-*O*-2′-glucuronide (*c*-ORV-2′G), which derived from their corresponding *trans* forms. In samples, the presence of *cis*-ORV-3-*O*-glucuronide was significant, while the quantities of *c*-ORV-2′G formed were very low (Figure 3). Note that neither the *trans* nor the *cis* form of ORV-4′G was detected in the samples. Moreover, the metabolization of ORV by human and rat enzymes was different. In fact, human enzymes add a glucuronide moiety preferably at the 2′ position, whereas rat enzymes do it at the 3 position (Figure 3). This interspecies difference was previously observed for ORV using human liver and intestinal microsomes.^27^ The authors also observed that ORV was glucuronidated mainly at the 2′ position. Mei and co-workers found that the 3 position was more common when ORV was metabolized with liver S9 and microsomes obtained from healthy Sprague Dawley rats.^28^

Concerning the *in vitro* production of GN glucuronides, two main metabolites were formed: *trans*-GN-2′-*O*-glucuronide (*t*-GN-2′G) and *trans*-GN-3-*O*-glucuronide (*t*-GN-3G) (the same as those previously synthesized) (Figure 3). Like ORV, rat enzymes metabolized GN at the 3 position and human enzymes metabolized GN at the 2′ position. *t*-GN-3G was identified in human enzymes, but the very low quantities produced ruled out their quantification. Unlike ORV, no isomerization was observed for GN, perhaps as a result of the difference in the positions of their hydroxyl groups. In fact, GN had a hydroxyl group at the 6′ position (and not ORV) that might induce steric hindrance, which would explain the greater tendency of *t*-ORV to isomerization. Overall, we present for the first time the *in vitro* metabolization of GN and the presence of two monoglucuronide metabolites: *t*-GN-2′G and *t*-GN-3G. Until now, the only work on the bioavailability of GN in rat established the presence of GN as a parent compound and metabolite in urine, without specifying the glucuronide position.^2^

**Figure 4:**
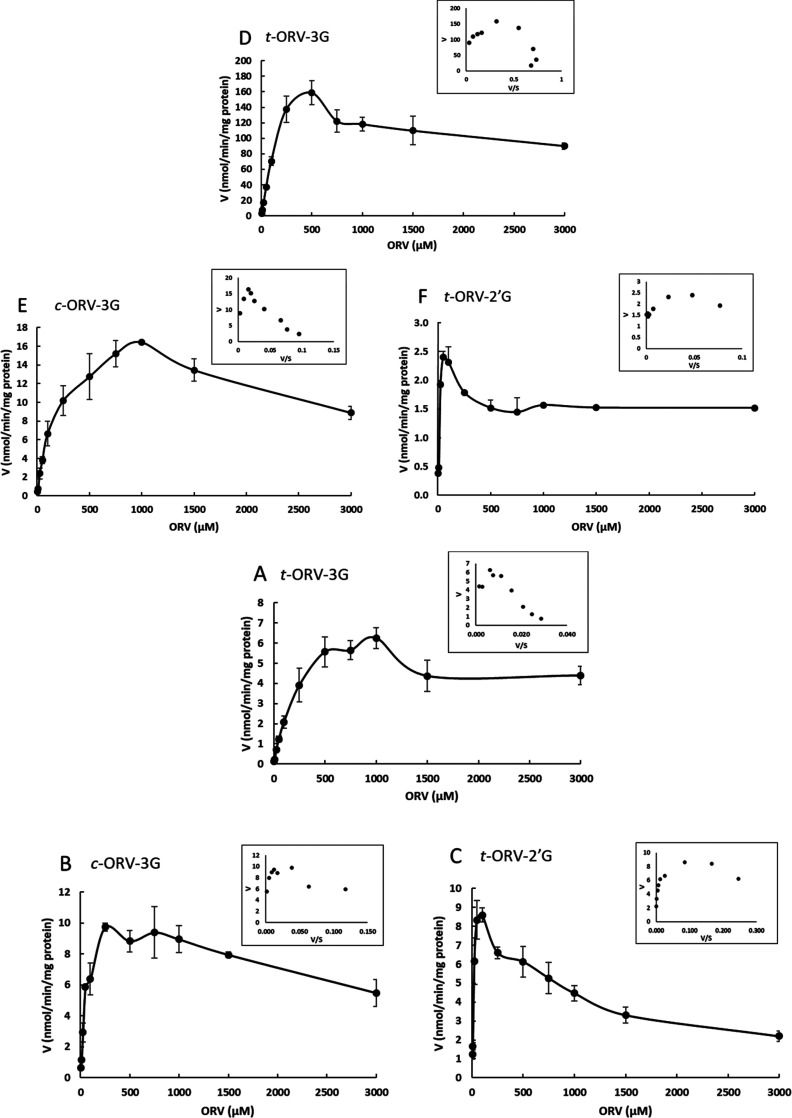
Kinetic profiles of formation of ORV metabolites by (A–C) human and (D–F) rat S9 fractions (0.5 mg/mL) incubated with ORV at different concentrations (0–3000 μM) in Tris–HCl buffer (50 mM, pH 7.4) in the presence of UDPGA (1 mM), alamethicin (25 μg/mL), and MgCl_2_ (5 mM). *n* = 4 (each experiment performed in triplicate separately).

The kinetic profiles of the formation of ORV glucuronide metabolites by human and rat S9 fractions are displayed in Figure 4 (nmol min^–1^ mg^–1^ of protein versus concentration) within their corresponding Eadie–Hofstee plots as insets. As observed, the formation of metabolites presents a clear substrate inhibition profile that was confirmed by the correlation coefficients (*r*^2^) calculated by fitting the obtained data to the substrate inhibition equation and by the direct visualization of a hook shaped on the Eadie–Hofstee plots (Table 2 and Figure 4). This type of profile has also been previously reported for ORV,^27^ resveratrol,^29^ and other stilbene monomers, such as piceatannol.^30^ Enzyme kinetic parameters [Michaelis constant (*K*_m_), maximal velocity of formation (*V*_max_), and *K*_i_ (μM)] of ORV glucuronidation by human and rat enzymes are displayed in Table 2. Briefly, *K*_m_ is the concentration of substrate required for the enzyme to achieve half *V*_max_. Thus, an enzyme with a high *K*_m_ has a low affinity for its substrate and requires a higher concentration of substrate to achieve *V*_max_. Concerning human metabolism, the highest *K*_m_ value was observed for *t*-ORV-3G (415.5 ± 142.9 μM) followed by their corresponding *cis* form (81.88 ± 15.84 μM) and *t*-ORV-2′G (32.65 ± 8.64 μM). *V*_max_ of the formation of these three metabolites was very similar. With regard to rat metabolism, the highest *K*_m_ values were also observed for *t*-ORV-3G and *c*-ORV-3G. However, the *V*_max_ values for these metabolites were quite different (Table 2). Considering the *V*_max_ values of ORV-3G for both species, they are considerably higher for rats, indicating that the catalytic potency of these enzymes in turning over the ORV to their glucuronide metabolites is more efficient in rats than in humans. Finally, according to the sum of the ratio *V*_max_/*K*_m_ (indicator of the intrinsic clearance over the intrinsic ability of hepatic enzymes to metabolize), rats have more than a 2-fold greater ability to metabolize ORV in its corresponding glucuronide metabolites than humans (1.284 versus 0.597 μL min^–1^ mg^–1^ of protein, for humans and rats, respectively) (Table 2).

**Table 2:** Kinetic Parameters of Glucuronidation of ORV and GN by Human and Rat Liver S9 Fractions

S9	ORV metabolite	*K*_m_ (μM)	*V*_max_ (nmol min^–1^ mg^–1^ of protein)	*K*_i_ (μM)	*V*_max_/*K*_m_ (μL min^–1^ mg^–1^ of protein)	sum of *V*_max_/*K*_m_	type of fit	goodness of fit (*r*^2^)
Human	*t*-ORV-3G	415.5 ± 142.9	11.41 ± 2.32	1712 ± 682.5	0.028 ± 0.016	0.597	SI	0.948
	*c*-ORV-3G	81.88 ± 15.84	13.01 ± 1.04	2403 ± 584.9	0.159 ± 0.066		SI	0.937
	*t*-ORV-2′G	32.65 ± 8.64	12.93 ± 1.47	486.3 ± 108.9	0.396 ± 0.170		SI	0.877
Rat	*t*-ORV-3G	319 ± 107.9	319.1 ± 69.77	860.9 ± 324.6	1.00 ± 0.646	1.284	SI	0.940
	*c*-ORV-3G	429.6 ± 125.9	31.34 ± 5.6	1440 ± 480	0.073 ± 0.044		SI	0.951
	*t*-ORV-2′G	16.82 ± 6.28	2.67 ± 0.32	1388 ± 537.7	0.159 ± 0.050		SI	0.651

S9	GN metabolite	*K*_m_ (μM)	*V*_max_ (nmol min^–1^ mg^–1^ of protein)	*K*_i_ (μM)	*V*_max_/*K*_m_ (μL min^–1^ mg^–1^ of protein)	sum of *V*_max_/*K*_m_	type of fit	goodness of fit (*r*^2^)
Human	*t*-GN-2′G	26.94 ± 7.65	2.48 ± 0.31	370.8 ± 88.02	0.092 ± 0.04	0.092	SI	0.85
Rat	*t*-GN-2′G	137.9 ± 30.1	6.88 ± 0.86	862.4 ± 204.5	0.050 ± 0.029	0.307	SI	0.95
	*t*-ORV-3G	139.9 ± 58.74	36 ± 3.57	2346 ± 610.2	0.257 ± 0.060		SI	0.94

**Figure 5:**
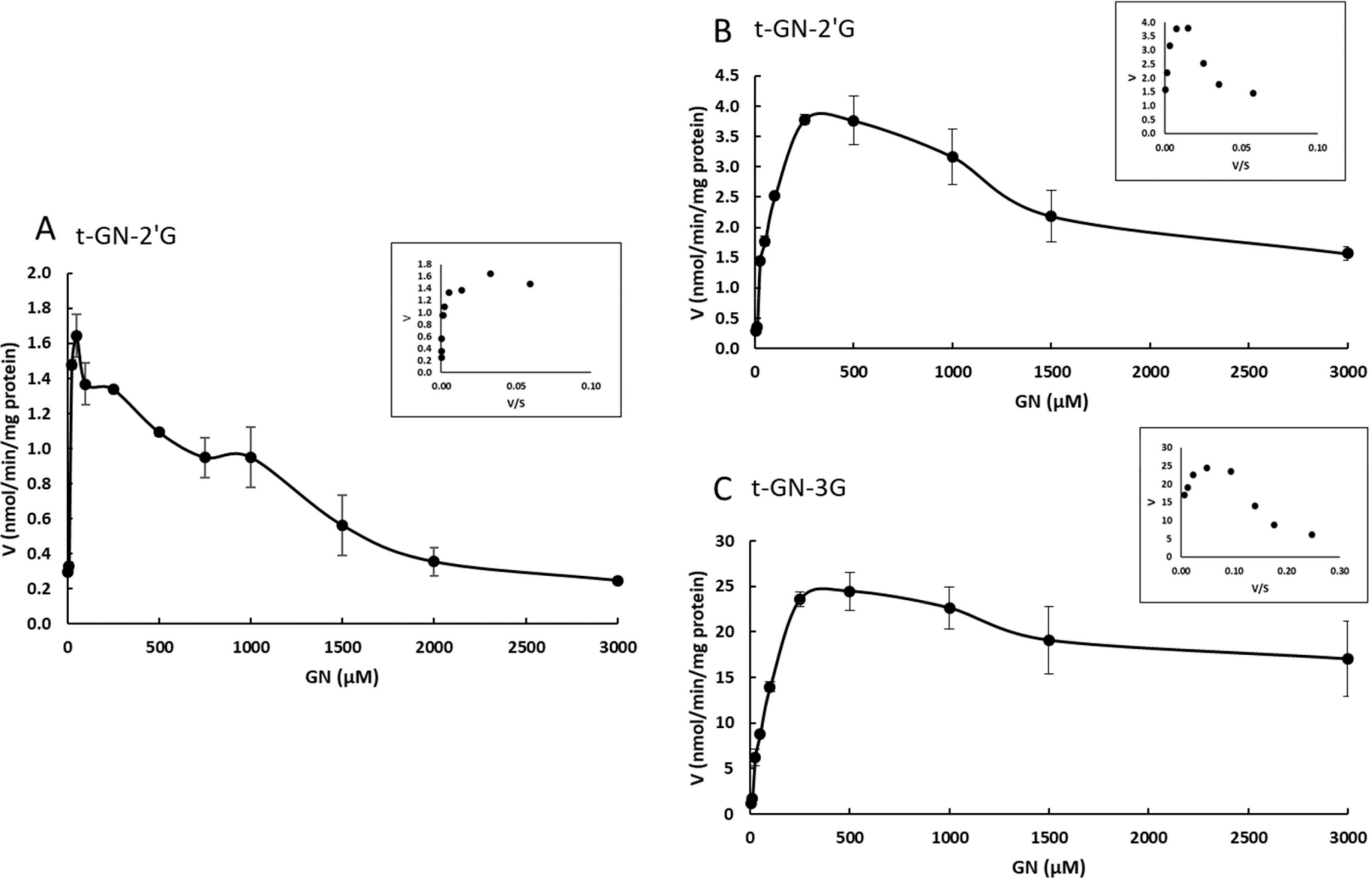
Kinetic profiles of formation of GN metabolites by (A) human and (B and C) rat S9 fractions (0.5 mg/mL) incubated with GN at different concentrations (0–3000 μM) in Tris–HCl buffer (50 mM, pH 7.4) in the presence of UDPGA (1 mM), alamethicin (25 μg/mL), and MgCl_2_ (5 mM). *n* = 4 (each experiment performed in triplicate separately).

Concerning the *in vitro* production of GN glucuronides, two main metabolites were formed: *t*-GN-2′G and *t*-GN-3G. As for ORV, the kinetic profiles of the formation of GN glucuronide metabolites in human and rat S9 hepatic fractions are summarized in Figure 5. A substrate inhibition profile can also be observed. With regard to GN, several differences between the species were observed. Only *t*-GN-2′G was produced by human hepatic enzymes, while both *t*-GN-2′G and *t*-GN-3G were observed after incubation with rat enzymes. Both *K*_m_ and *V*_max_ values were higher in rats. Like ORV, *V*_max_ values were higher in rats, thus proving its high potential for metabolization. With regard to *V*_max_/*K*_m_ (μL min^–1^ mg^–1^), the same trend as for ORV was noticed, with rats having a greater ability to metabolize GN in their corresponding glucuronide metabolites (0.092 and 0.307, for humans and rats, respectively). Our findings show that the mode of metabolizing ORV and GN varies according to the species. Human enzymes glucuronidated preferably at the 2′ position, while rat enzymes do so at the 3 position. This is probably due to differences in the expression of UGTs between rats and humans and also differences in their UGT activities. Indeed, in animal models, such as rats, mice, dogs, and monkeys, various UGT isoforms are expressed. Even though an animal isoform may be identified as a human orthologue, it can display a different substrate specificity and tissue distribution compared to the human isoform.^31^ A study found that the quantities of different glucuronide metabolites of resveratrol (3 and 4′) that are formed differ substantially depending upon the origin of the microsomes used: human, dogs, or rodents. For example, resveratrol-4′-*O*-glucuronide is formed when using human and dog microsomes, while rat microsomes do not form it.^32^ As mentioned above, ORV is known to be glucuronidated preferentially at the C-2 position in human liver and intestinal microsomes.^27^ However, the 3 position is preferred when it is metabolized with rat microsomes.^28^ Concerning GN, this is the first time that two monoglucuronide metabolites are unambiguously characterized after *in vitro* glucuronidation by human and rat enzymes.

### Anti-inflammatory Activity of ORV, GN, and Their Metabolites

Chronic inflammation and oxidative stress are pathological conditions that can lead to several disabling and fatal diseases, such as cardiovascular disease, cancer, diabetes mellitus, chronic kidney disease, non-alcoholic fatty liver disease, and autoimmune and neurodegenerative disorders.^33^ NO, ROS, IL-1β, and TNF-α levels are well-established mediators of the inflammatory and oxidative stress processes.^34^ The anti-inflammatory properties of ORV, GN, and their metabolites were assessed by measuring NO, ROS, IL-1β, and TNF-α in a macrophage model cell line (RAW 264.7 cells) stimulated with LPS (a bacterial cell wall product of Gram-negative bacteria), a well-known potent activator of inflammatory response in macrophages.

**Figure 6:**
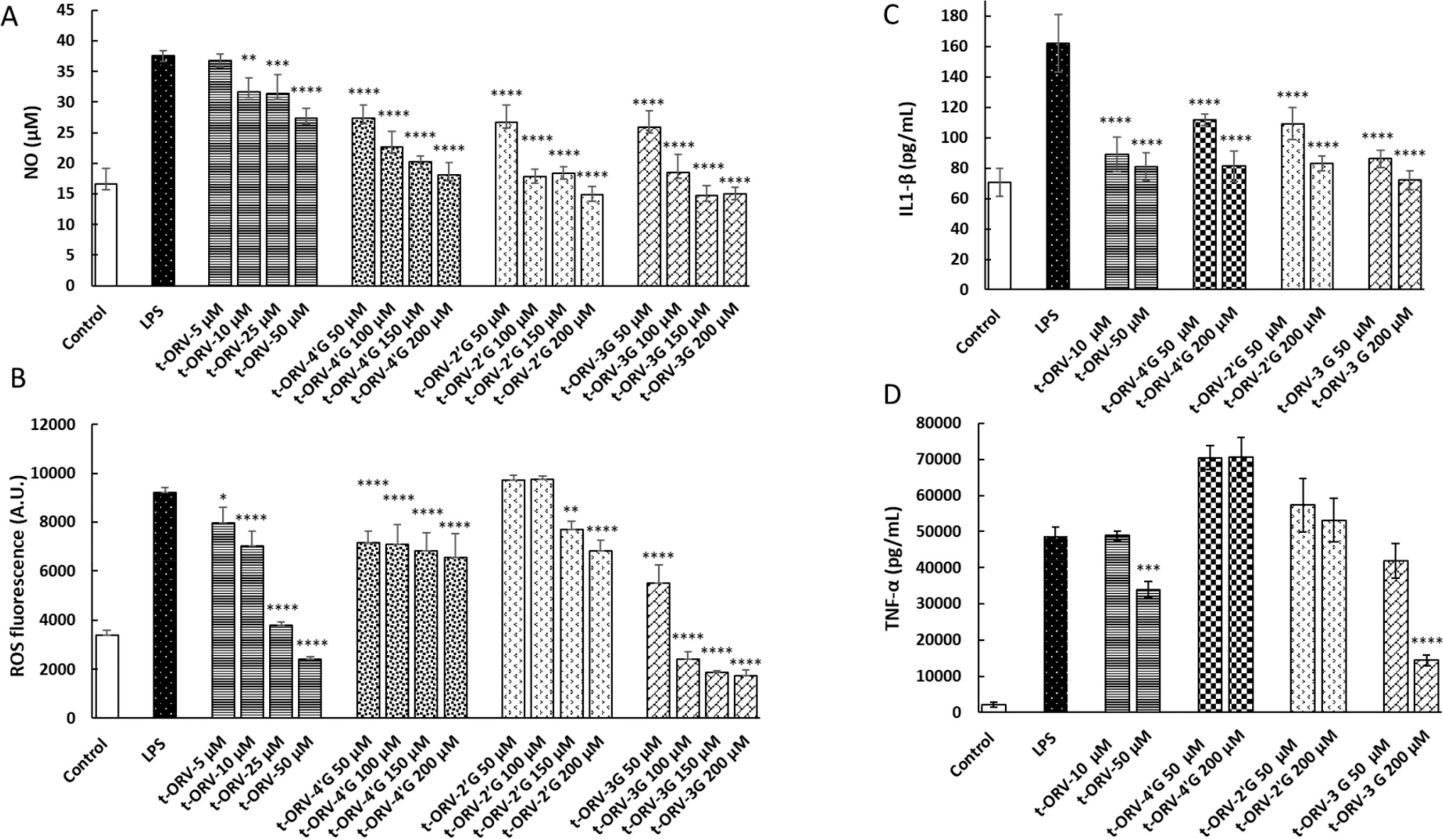
(A) NO (μM), (B) ROS production, (C) IL-1β (pg/mL), and (D) TNF-α (pg/mL) in RAW 264.7 cells. Cells were treated for 24 h by LPS (0.1 μg/mL) or LPS with *t*-ORV, *t*-ORV-4′G, and *t*-ORV-3G (5–200 μM). *n* = 4 (each experiment performed in triplicate separately).

Before evaluation of the anti-inflammatory potential of *t*-ORV, *t*-GN, and their metabolites, their cytotoxicity was studied using the MTT assay, which allows for the determination of metabolically active cells. Thus, RAW 264.7 cells were exposed to different concentrations of parent compounds and glucuronide metabolites (5–200 μM) for 24 h. Both *t*-ORV and *t*-GN were toxic from 100 μM, while all tested concentrations of metabolites were non-cytotoxic for the cells, even at the maximal concentration tested: 200 μM (Figure S1 of the Supporting Information). In RAW 264.7 cells, our research group has previously reported that IC_30_ for cell cytotoxicity of *t*-ORV is higher than 50 μM; this result is in accordance with the observations in the present paper.^35^ On the other hand, other authors did not observe any cytotoxic effect at 100 μM,^6^ although differences in incubation times (18 versus 24 h) might explain this discrepancy. Hankittichai and co-workers also observed that *t*-ORV begins to be toxic to HMC3 cells from an 80 μM concentration.^8^ Concerning *t*-GN, the lack of data in macrophages does not enable us to compare our results. However, no cytotoxic effect was observed at 5 and 10 μM in BV-2 microglial cells.^36^ With regard to *t*-ORV and *t*-GN glucuronide metabolites, any study has been conducted; however, neither resveratrol-3-*O*-glucuronide nor resveratrol-4′-*O*-glucuronide had any cytotoxic effect on macrophages.^37^ Therefore, the concentrations used to study their anti-inflammatory properties were chosen on the basis of our results on cell viability and sparse data of plasma concentrations of *t*-ORV and *t*-GN.^17−20^ Thus, we used concentrations between 5 and 50 μM for *t*-ORV and *t*-GN and between 50 and 200 μM for glucuronide metabolites, which are generally present in higher quantities in plasma.^21,22^Figure 6A shows NO cell production (μM) in cells treated with LPS alone or with LPS and *t*-ORV and its metabolites (*t*-OXY-4′G, *t*-OXV-2′G, and *t*-OXV-3G). As expected, the NO concentration of the positive control (cells treated with LPS) increased more than 2-fold in comparison to the negative control without LPS treatment (Figure 6A). In addition, *t*-ORV significantly reduced the production of NO from 10 μM to achieve a reduction of 27.1% at 50 μM. With regard to glucuronide metabolites, the same effect was noticeable at this concentration, with a 27.2, 29, and 31% reduction of *t*-OXY-4′G, *t*-OXV-2′G, and *t*-OXV-3G at 50 μM, respectively.

Furthermore, our results demonstrate that *t*-ORV is a potent antioxidant against intracellular ROS production induced by LPS (Figure 6B). *t*-ORV reduced more than 70% of the ROS at 50 μM. Some differences were found with ORV metabolites. Whereas *t*-ORV-4′G and *t*-ORV-2′G can diminish the production of ROS by between 22 and 25% at the most, a much higher effect was observed for *t*-ORV-3G, which can reduce it by more than 80% (Figure 6B).

To gain insight into the release of inflammatory mediators, IL-1β and TNF-α concentrations (pg/mL) were measured in culture medium of macrophages treated with *t*-ORV (10 and 50 μM) and *t*-OXY-4′G, *t*-OXV-2′G, and *t*-OXV-3G (50 and 200 μM). We used low and high concentrations based on the previous results concerning NO and ROS to investigate a possible dose–response effect. All stilbenes reduced the release of IL-1β (between 30 and 50%). Note that to obtain the same effect as the parent compounds, a 4-fold concentration of glucuronide metabolites is needed. A slightly more potent effect was observed with *t*-ORV glucuronide at the 3 position (Figure 6C). Nevertheless, *t*-ORV and *t*-ORV-3G were the only compounds that significantly reduced TNF-α at 50 μM (30%) and 200 μM (70%), respectively (Figure 6D). These results confirm that glucuronidation at the 3 position or glucuronidation on the A cycle of the stilbene instead of the B cycle affects the bioactivity of the molecule. The results obtained for *t*-ORV are in accordance with data published very recently demonstrating that, at the same concentrations, it is able to suppress the release of not only NO, TNF-α, and IL-1β but also iNOS, MCP-1, CXCL10, and IL-6 in macrophages and microglial cells.^7,8^ However, we are the first to report the anti-inflammatory activity of *t*-ORV glucuronide metabolites.

**Figure 7:**
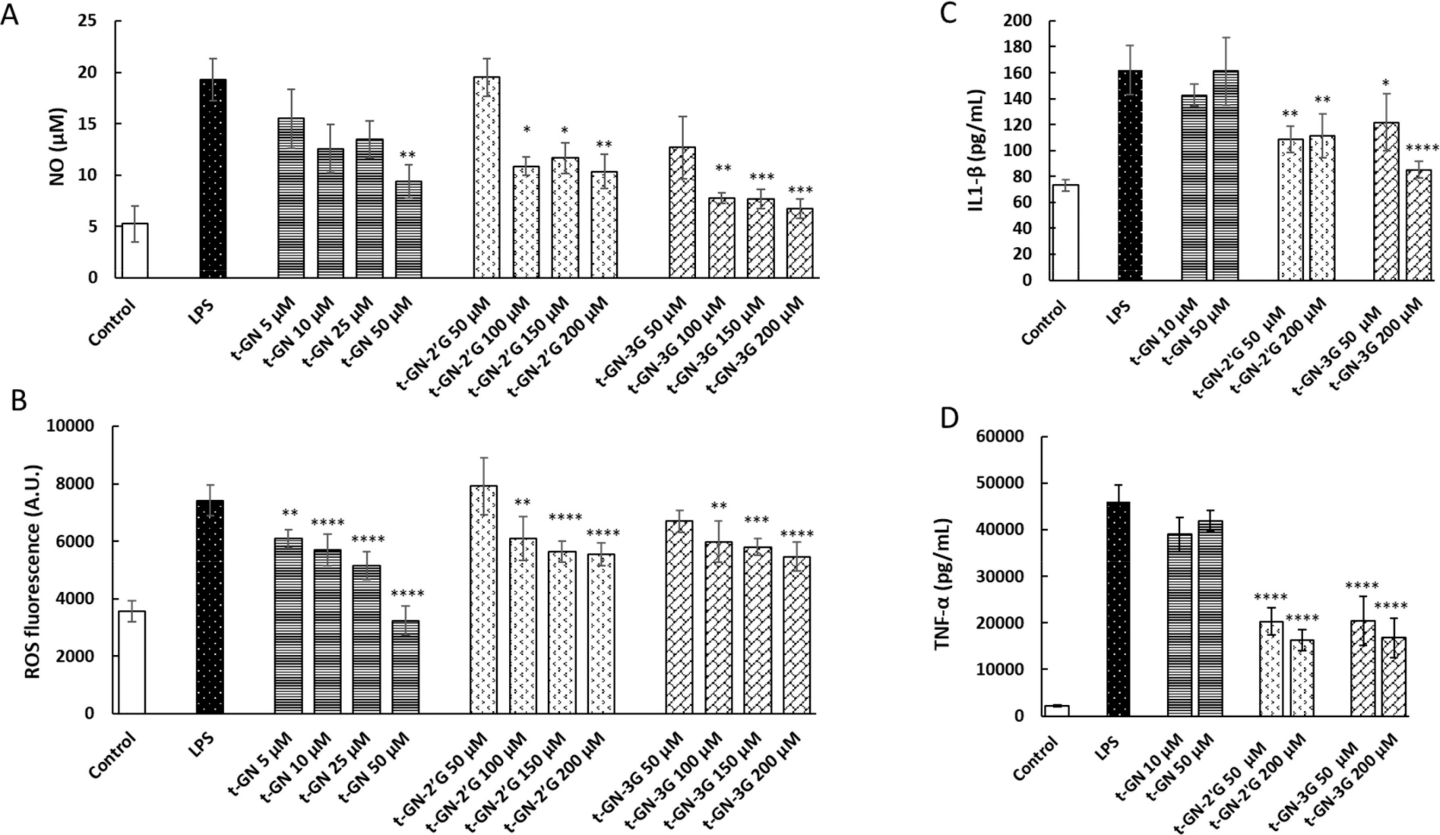
(A) Cell viability (%), (B) NO (μM) and ROS production, (C) IL-1β (pg/mL), and (D) TNF-α (pg/mL) in RAW 264.7 cells. Cells were treated for 24 h by LPS (0.1 μg/mL) or LPS with *t*-GN, *t*-GN-2′G, and *t*-GN-3G (5–200 μM). *n* = 4 (each experiment performed in triplicate separately).

Next, the anti-inflammatory activity of *t*-GN and its metabolites (*t*-GN-2′G and *t*-GN-3G) was assessed. At the highest concentration tested, they decreased the production of NO by between 46 and 65% (Figure 7A). Again, the most potent effect was obtained with GN glucuronide at the 3 position on the A cycle. With regard to ROS, GN had the stronger effect, counteracting ROS production by more than 55% (Figure 7B). Interestingly, even though their effect on ROS was slight, both metabolites significantly reduced IL1-β (32–47%) and TNF-α (around 65%), whereas *t*-GN was ineffective (panels C and D of Figure 7). Therefore, *t*-GN and its metabolites can potentially limit the release of NO, cytokines, and ROS. Resveratrol and its metabolites (glucuronides and sulfates) have been widely studied for their potential anti-inflammatory properties. For example, Walker and collaborators demonstrated that the position of the glucuronide moiety affects their bioactivity, in agreement with our findings. Resveratrol-4′-*O*-glucuronide upregulated mRNA levels of macrophage inflammatory protein 1β (MIP-1β), whereas resveratrol-3-*O*-glucuronide did not have this effect in LPS-activated U-937 macrophages.^38^

With regard to the anti-inflammatory and antioxidant activities of both stilbenes and their corresponding metabolites, it can be concluded that *t*-ORV and its metabolites generally have a greater effect than GN and its glucuronides. In addition, the position of the glucuronide moiety affects the bioactivity of the molecule.

To summarize, the hemisynthesis of three ORV glucuronides and two GN glucuronides allowed us to study the hepatic metabolism of these two stilbenes *in vitro*. Several differences were found between humans and rats. Human enzymes glucuronidated preferably at the 2′ position, whereas rat enzymes do so at the 3 position. Given the kinetic parameters (*K*_m_, *V*_max_, and *V*_max_/*K*_m_), rat enzymes have a stronger metabolic capacity than human enzymes. We report for the first time that *t*-ORV, *t*-GN (between 25 and 50 μM), and mainly the metabolites glucuronidated at the 3 position (150 and 200 μM) are able to decrease NO and ROS production in LPS-stimulated murine macrophages by more than 50%. Additionally, *t*-ORV, *t*-GN, and their metabolites limit the production of IL-1β and TNF-α mediators. Even though larger concentrations of glucuronide metabolites are needed to obtain the effect, the fact that they are present in greater quantities in biological fluids than their parent compounds is of considerable biological importance.

## Supporting Information Available

Cell viability of *t*-ORV, *t*-GN, and their glucuronide metabolites (Figure S1) (PDF)

## Abbreviations Used

*c*-ORV-2′G: *cis*-oxyresveratrol-2′-*O*-glucuronide
*c*-ORV-3G: *cis*-oxyresveratrol-3-*O*-glucuronide
IL-1β: interleukin 1β
NO: nitric oxide
ROS: reactive oxygen species
*t*-ORV: *trans*-oxyresveratrol
*t*-ORV-2′G: *trans*-oxyresveratrol-2′-*O*-glucuronide
*t*-ORV-3G: *trans*-oxyresveratrol-3-*O*-glucuronide
*t*-ORV-4′G: *trans*-oxyresveratrol-4′-*O*-glucuronide
*t*-GN: *trans*-gnetol
*t*-GN-2′G: *trans*-gnetol-2′-*O*-glucuronide
*t*-GN-3G: *trans*-gnetol-3-*O*-glucuronide
TNF-α: tumor necrosis factor α
