# Supplementary material for: Oxyresveratrol and Gnetol Glucuronide Metabolites: Chemical Production, Structural Identification, Metabolism by Human and Rat Liver Fractions, and In Vitro Anti-inflammatory Properties
Source: J Agric Food Chem. 2022 Feb 23;70(41):13082–92. doi: 10.1021/acs.jafc.1c07831 (PMC9585577; doi:10.1021/acs.jafc.1c07831)
Supplement: Supplementary file 1 — jf1c07831_si_001.pdf [file jf1c07831_si_001.pdf]

Supplementary Material

Oxyresveratrol and gnetol glucuronide metabolites: chemical production, structural identification, metabolism by human and rat liver fractions and *in vitro* anti-inflammatory properties

Ruth Hornedo-Ortega,* Michaël Jourdes, Gregory Da Costa, Arnaud Courtois, Julien Gabaston, Pierre-Louis Teissedre, Tristan Richard and Stéphanie Krisa

Unité de Recherche Œnologie, Institut des Sciences de la Vigne et du Vin, Université de Bordeaux. EA 4577, USC 1366 INRAe, IPB. 210, chemin de Leysotte CS 50008. 33882 Villenave d'Ornon cedex. France

*Corresponding author: rhornedo@us.es

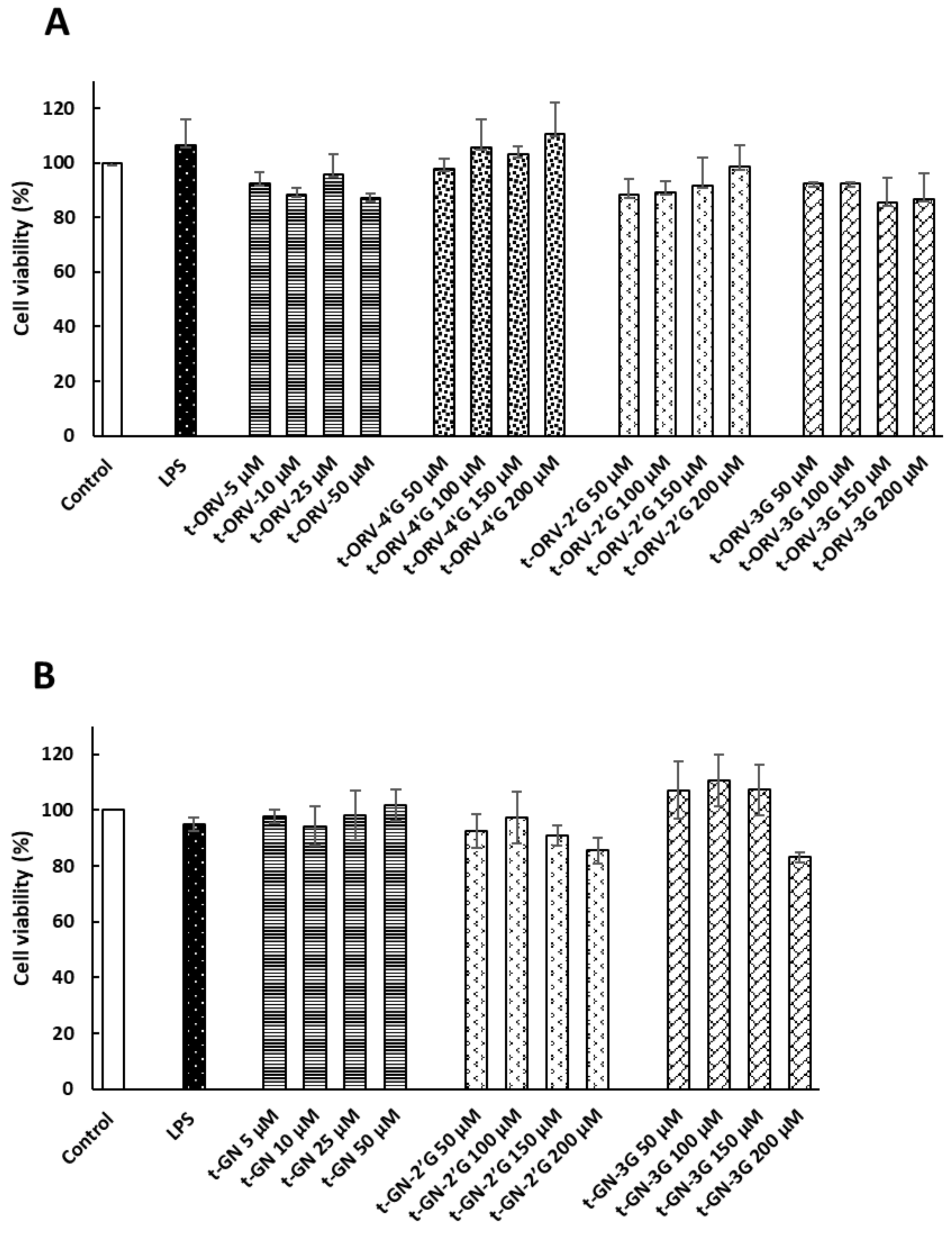

Figure S1. Cell viability (%) of *t*-ORV (5-50 µM) and their glucuronide metabolites (50-200 µM) (A) and of *t*-GN (5-50 µM) and their glucuronide metabolites (50-200 µM) (B) in RAW264.7 macrophages at different concentrations.
